# Malocclusion Among 10- to 12-Year-Old Male Schoolchildren in Qassim Region of Saudi Arabia: A Retrospective Epidemiological Study

**DOI:** 10.7759/cureus.20459

**Published:** 2021-12-16

**Authors:** Abdulaziz N Alajaji, Rakan S Alogaili, Zeyad Alsughier

**Affiliations:** 1 Dentistry, Qassim University, Buraydah, SAU

**Keywords:** qassim, saudi arabia, schoolchildren, prevalence, malocclusion

## Abstract

Objective

To identify the prevalence of malocclusion in late mixed dentition in Qassim region of Saudi Arabia. This will be the first epidemiological study of its kind in this region. It will be very helpful for planning effective preventive measures and therapy programs.

Materials and methods

This study was performed in Qassim region, Saudi Arabia starting from October 2018 to March 2019. The examination was performed by two well-trained general dentists after using a specially prepared clinical examination form. A total of 536 children aged between 10 and 12 and those who met the inclusion criteria have been examined for Angle’s relationship, overjet, overbite, crossbite, midline deviation and lip competent.

Results

Class I relation accounted for the highest percentage of the sample, whilst 31.3% presented with Class I ideal occlusion, and 48.9% Class I with malocclusion. This was followed by Class II malocclusion (12.5% of the sample), and Class III accounted for the lowest proportion (7.3%). Increased overjet was present in 34.4% of the sample, whereas 3.9% had edge-to-edge and 2.2% a reverse overjet. Regarding overbite, 39% reported increased overbite, whilst 3% had open bite. A total of 63 children presented with crossbite - 6.15% had anterior crossbite, 5% unilateral posterior, and 0.5% bilateral posterior. Regarding the midline, only visible and noticed deviation was recorded. The results showed that 90% had no deviation, while 10% had a deviated midline. Regarding lip competence, only 12.1% had an incompetent lip.

Conclusion

Early intervention and correction of occlusal discrepancies will facilitate the treatment and eliminate possible defects in developing dental arches.

## Introduction

Besides dental caries, malocclusion is considered one of the most common dental diseases, in addition to periodontal disease and dental fluorosis [1,2]. A malocclusion is defined as an improper alignment of the teeth or the deviation of molar relationship between upper and lower dental arches from the normal ideal position, which is unsatisfactory aesthetically and functionally [3]. Abnormal occlusion may cause an unfavorable appearance, disrupted oral function, speech difficulties, temporomandibular joint diseases, and contribute in dental trauma and periodontal disease as a result of food impaction and difficulty of teeth brushing [1,4]. Assessment of occlusal status in a specific population and collecting data related to the prevalence of each occlusal traits and its age distribution will provide important information about the treatment needed and allow the government to formulate appropriate preventive and treatment programs and to prevent further progression of malocclusion by early intervention [5]. Early intervention in children with severe Class II malocclusion and increased overjet may decrease susceptibility to tooth trauma by fall or other sport-related injuries. Data on the incidence and progression of each occlusal traits, together with information on the validity of treatment needs are required for estimating the need for early intervention [2]. The disadvantage of an early intervention is the long duration of treatment with reduced compliance and unpredictable growth pattern [4]. The prevalence of malocclusion in various races and populations during the mixed dentition stage was studied in several previous researches [6-13]. The variability between different studies due to variation in identification patterns of different classes of occlusal relationship, examiner variation in interpretation of each type of malocclusion and the differences between sample size for each population may have an impact on the result. According to our literature review using Pubmed and Google Scholar to search for articles discussing the prevalence of malocclusion among schoolchildren, this will be the first epidemiological study of its kind in this region of Saudi Arabia. It will be very helpful for planning effective preventive measures and therapy programs.

The aim of this study was to determine the prevalence of malocclusion among male school children aged 10-12 years in Qassim region, Saudi Arabia, and to estimate the proportion of each form of malocclusion in order to establish treatment and prevention programs for early intervention.

## Materials and methods

A cross-sectional study has been performed among 10-12 years old male school children to estimate the prevalence of malocclusion in Qassim region, Saudi Arabia, from October 2018 to March 2019. The school was a unisex (male only) school and that is why we limited our research to male children. Ethical approval was obtained from the Dental Research Center, College of Dentistry, Qassim University with approval number: DEC-1/7005/2018 dated 17/10/2018. Six schools were randomly selected from different geographic locations (Buraidah, Unaizah, Al Khabra, Al Badayea, and Al Bukairiyah). Approval was obtained from the individual head of schools and a consent letter was sent to each student’s parents or guardian. The inclusion criteria were as follows: child’s age between 10 and 12 years, all the permanent first molars have erupted. The exclusion criteria were as follows: very uncooperative child, any history of orthodontic treatment, craniofacial anomalies. A total of 545 male schoolchildren from different public schools were examined and screened, 536 out of 545 students met the inclusion criteria.

Clinical examination

The examination was done by two well-trained general dentists after receiving a parental consent form for each child enrolled in the study. The examination was carried out by using disposable gloves, a gown, mask, tongue depressor, sterilized ruler and UNC-15 probes. While the child was sitting on a chair with a normal natural light in the room, one of the examiners did screening and the other was responsible for data collection. The following information was collected from the sample during screening: Angle’s relationship in centric occlusion position, we tried to get the most accurate reading by asking the patient to swallow and then bite using his most posterior teeth. Overjet and overbite were measured using a sterilized ruler and UNC-15 probes. The transverse relationship was evaluated by cheek retraction and inspection for the following: unilateral posterior crossbite, bilateral posterior crossbite and anterior crossbite. Midline deviation and lip competence were examined and recorded. Oral photos were taken for each child with severe malocclusion to be used as a record. Any child requiring dental treatment was referred to the dental clinic at Qassim University.

Statistical analysis

Study data was analysed using the Chi-Square test. We used the statistical software SPSS version 21 (IBM Corp., Armonk, NY).

## Results

**Figure 1:**
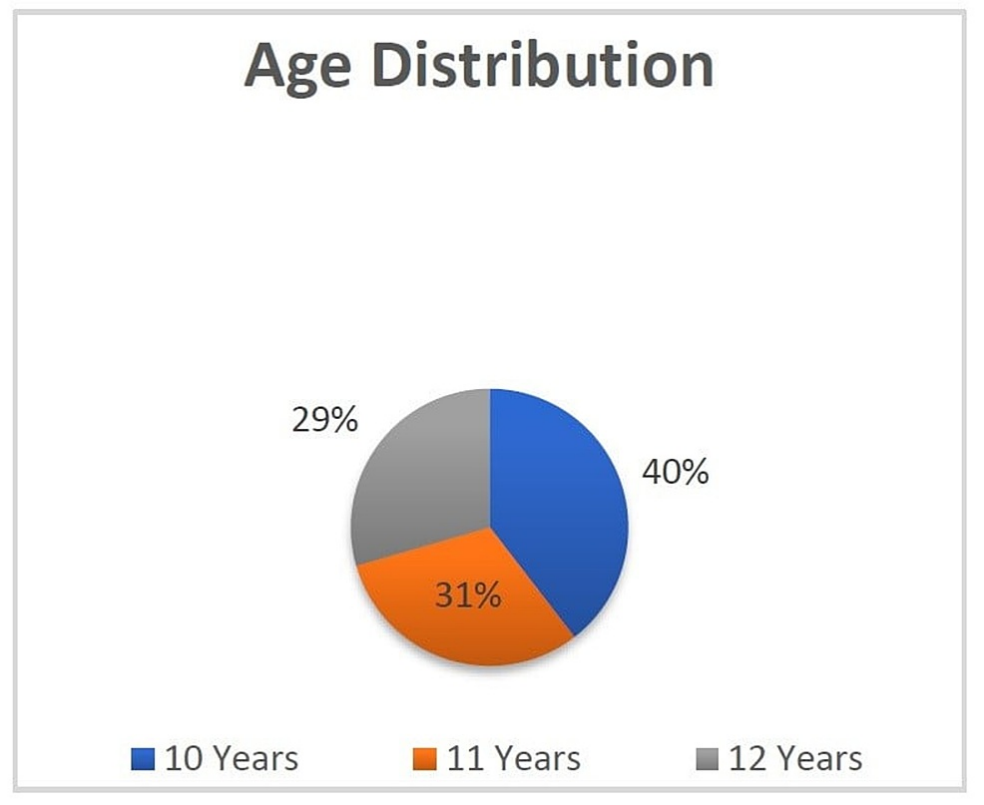
Age distribution in the study.

The age distribution of the 536 children examined was as follows: 40% were 10 years old, 31% 11 years old and 29% 12 years old (Figure 1).

In correspondence to Angle’s classification, 31.3% presented with Class I normal occlusion, 48.9% of children presented with Class I malocclusion; Class I relation considered as the highest percentage between the other readings. Class II malocclusion involved only 12.5% of the sample. Within Class II malocclusion, 8.6% of children were division 1 type and 3.9% division 2 type. Class III malocclusion was the lowest reading with only 7.3% of the sample (Table 1).

**Table 1:** Classification of the subjects based on Angle’s classification of occlusion.

		Age (years)		
Occlusion	10 N (%)	11 N (%)	12 N (%)	Total
Normal occlusion	70 (41.7%)	51 (30.4%)	47 (28.0%)	168 (31.3%)
Class I malocclusion	93 (35.5%)	85 (32.4%)	84 (32.1%)	262 (48.9%)
Class II div I	20 (43.5%)	12 (26.1%)	14 (30.4%)	46 (8.6%)
Class II div II	11 (52.4%)	6 (28.6%)	4 (19.0%)	21 (3.9%)
Class III malocclusion	18 (46.2%)	12 (30.8%)	9 (23.1%)	39 (7.3%)
Total	212 (39.6%)	166 (31.0%)	158 (29.5%)	536 (100.0%)
Chi-square = 5.413, P = 0.713				

Of the 536 children who were examined for overjet, 59.5% had normal ≤3mm overjet, and 22.6% had slightly increased overjet, while 11.8% had severely increased overjet. Among the screened children 3.9% had edge-to-edge. A small percentage of the sample with only 2.2% had reverse overjet (Table 2).

**Table 2:** Distribution of overjet among the subjects.

		Age (years)		
Overjet	10 N (%)	11 N (%)	12 N (%)	Total
Normal ≤ 3mm	128 (40.1%)	97 (30.4%)	94 (29.5%)	319 (59.5%)
Edge to edge	9 (42.9%)	6 (28.6%)	6 (28.6%)	21 (3.9%)
<3mm	43 (35.5%)	39 (32.2%)	39 (32.2%)	121 (22.6%)
≥6mm	26 (41.3%)	21 (33.3%)	16 (25.4%)	63 (11.8%)
Reverse overjet	6 (50.0%)	3 (25.0%)	3 (25.0%)	12 (2.2%)
Total	212 (39.6%)	166 (31.0%)	158 (29.5%)	536 (100.0%)
Chi-square = 2.096, P = 0.978				

Regarding overbite, 54.3% had normal ≤3mm overbite, 3.9% had edge-to-edge, 39% had increased <3mm overbite and a small percentage (3%) had open bite (Table 3).

**Table 3:** Distribution of overbite among the subjects.

		Age (years)		
Overbite	10 N (%)	11 N (%)	12 N (%)	Total
Normal ≤ 3mm	118 (40.5%)	89 (30.6%)	84 (28.9%)	291 (54.3%)
Edge to edge	8 (39%)	7 (33.5%)	6 (28%)	21 (3.9%)
Increased < 3mm	77 (36.9%)	665 (31.1%)	66 (31.6%)	208 (39%)
Open bite	9 (56.2%)	5 (31.2%)	2 (12.5%)	16 (3.0%)
Total	212 (39.6%)	166 (31.0%)	158 (29.5%)	536 (100.0%)
Chi-square = 3.603, P = 0.730				

To investigate the transverse relationship between the examined children, a total of 63 children presented with crossbite, of which 33 (52.4%) had anterior crossbite (single or two teeth). The most commonly seen teeth were the maxillary central and lateral incisors, and 27 (42.9%) of the sample had unilateral posterior crossbite. Three children (4.8%) had bilateral posterior crossbite (Table 4).

**Table 4:** Crossbite distribution among the subjects.

		Age (years)		
Crossbite	10 N (%)	11 N (%)	12 N (%)	Total
Anterior crossbite (single or 2 teeth)	14 (42.4%)	11 (33.3%)	8 (24.2%)	33 (52.4%)
Unilateral posterior crossbite	8 (29.6%)	10 (37.0%)	9 (33.3%)	27 (42.9%)
Bilateral posterior crossbite	1 (33.3%)	0 (0.0%)	2 (66.7%)	3 (4.8%)
Total	23 (36.5%)	21 (33.3%)	19 (30.2%)	63 (100.0%)
Chi-square = 3.585, P = 0.465				

In the examination of midline, only the visible and noticed deviation was recorded; slight deviation was considered as normal. According to this, 90.7% of the children had no deviation, while 9.4% had deviation, 4.9% deviated to the right and 4.5% deviated to the left (Table 5).

**Table 5:** Distribution of midline deviation among the subjects.

		Age (years)		
Midline deviation	10 N (%)	11 N (%)	12 N (%)	Total
No deviation	194 (39.9%)	150 (30.9%)	142 (29.2%)	486 (90.7%)
Deviated to the right	8 (30.8%)	9 (34.6%)	9 (34.6%)	26 (4.9%)
Deviated to the left	10 (41.7%)	7 (29.2%)	7 (29.2%)	24 (4.5%)
Total	212 (39.6%)	166 (31.0%)	158 (29.5%)	536 (100.0%)
Chi-square = 0.934, P = 0.920				

Regarding lip competence, 87.9% had normal competent lip, while 12.1% had incompetent lip (Table 6).

**Table 6:** Distribution of lip competence among the subjects.

		Age (years)		
Lip competence	10 N (%)	11 N (%)	12 N (%)	Total
Competent	181 (38.4%)	150 (31.8%)	140 (29.7%)	471 (87.9%)
Incompetent	31 (47.7%)	16 (24.6%)	18 (27.7%)	65 (12.1%)
Total	212 (39.6%)	166 (31.0%)	158 (29.5%)	536 (100.0%)
Chi-square = 2.284, P = 0.319				

## Discussion

In this study, the prevalence of Class I occlusion was 80.2% (31.4% normal ideal occlusion and 48.9% Class I with visible malocclusion). Higher percentages were found among other studies such as the study from Tanzania [1], while lower percentages were found in Riyadh City, Saudi Arabia [5,13], Kuwait [12] and Jordan [11]. As for Class II, it accounted for 12.5% of the sample (8.6% Division I and 3.9% Division II). Lower values (4.4%) were found in the Tanzanian population [1], while higher values were found in Riyadh City, Saudi Arabia [5,13], Kuwait [12], Jordan [11] and Iran [14]. Class III accounted for only 7.3% of children in this study, a similar figure to the studies carried out in Iran [14] and Riyadh, Saudi Arabia [13].

However, lower values were found in Kerala, South India [15], and Tanzania [1]. On the other hand, higher values were found in Kuwait [12] and Jordan [11].

In the current study, overjet less than or equal to 3mm was considered as normal. A total of 59.5% of the subjects were therefore considered normal, which corresponds to the findings reported for Kuwaitis [12] and Jordanians [11]. Higher values were found in Saudi Arabia [5,13], India [16], Iran [14] and Tanzania [1]. A total of 22.6% of the subjects had a moderate increase in overjet that measured more than 3mm and less than 6mm, a similar result to that reported in Jordan [11], and Iran [14]. Lower values were found in Saudi Arabia [5,13], and higher values in India [15] and Kuwait [12]. Severely increased overjet was present in 11.8% of the subjects, which is similar to the findings from Tanzania [1]. Edge-to-edge relation was present in 3.9% of the subjects, which is consistent with the findings seen in studies done in Kuwait [12] and Iran [14]. Reverse overjet was present in 2.2% of the subjects, which was similar to the findings seen in the study done in Riyadh [13].

The prevalence of normal overbite was consistent with findings in Iran [14]. On the contrary, higher prevalence rates were found in Tanzania [1], and India [15]. A total of 39% of the subjects had increased overbite, which corresponds with the study findings from India [15]. Open bite was seen in 3% of the sample, a similar proportion was found in previous studies done in Saudi Arabia [13], Kuwait [12], Sweden [7,8] and Jordan [11], while a lower proportion was found in India [15].

Regarding crossbite, only 63 (12%) subjects of the total sample presented with different types of crossbite. Anterior crossbite, either a single or two teeth, was seen in 52.4% of them, while 42.9% presented with unilateral posterior crossbite. A lower percentage (4.8%) had a bilateral posterior crossbite. Higher prevalence rates of bilateral posterior crossbite were seen in Iran [14] and Kuwait [12], while lower prevalence was seen in India [15], Jordan [11] and Tanzania [1].

For midline deviation, slight deviation was not recorded, and only visible deviation was considered. In this study, only 10% of subjects presented with midline deviation. A number of studies - Abu Alhaija et al. [11], Narayanan et al. [15] and Mtaya et al. [1] - reported a higher prevalence of midline deviation. The variation in the results may be attributed to differences in registration methods. The prevalence of incompetent lip in Qassim schoolchildren was similar to that reported in Tausche et al. [16].

Our study was limited by the fact that it was done in single-sex schools as there are no mixed schools in Saudi Arabia. Our future plan is to do another study in female schools to compare male and female school children regarding malocclusion.

## Conclusions

Prevalence of the different anomalies between male schoolchildren in Qassim is comparable with that in other studies carried out in different countries. Class I malocclusion was the most frequent anomaly seen in our study population and the prevalence of this type of malocclusion was higher in our study than in most of the other studies in the middle east. Increased overjet and increased overbite were the other common frequent occlusal disharmonies found in this study. Early intervention and correction of occlusal discrepancies will facilitate the treatment and eliminate possible defects in developing dental arches. The findings of this study provide helpful data to use in early interceptive treatment programs and promote awareness among parents, pediatricians and general dentists.
